# Immune cell β2-adrenergic receptor signaling in myocardial injury: from neuroimmune crosstalk to precision immunomodulation

**DOI:** 10.3389/fimmu.2026.1976748

**Published:** 2026-10-26

**Authors:** Xiaolin Su, Lin Li, Weizhong Han, Yan Hao

**Affiliations:** 1 Department of Cardiology, Shandong Provincial Hospital Affiliated to Shandong First Medical University, Jinan, Shandong, China; 2 Shandong First Medical University, Jinan, Shandong, China

**Keywords:** immune cells, inflammatory response, myocardial injury, therapeutic strategies, ventricular remodeling, β2-adrenergic receptor

## Abstract

The β2-adrenergic receptor (β2-AR), a G protein-coupled receptor widely expressed on immune cells, has emerged as an important regulator linking sympathetic activation with inflammatory responses during myocardial injury. Beyond its established cardiovascular functions, β2-AR orchestrates immune cell migration, polarization, cytokine production, and efferocytosis, thereby shaping the initiation, resolution, and repair phases of cardiac inflammation. Recent studies have identified several immune-specific mechanisms through which β2-AR regulates leukocyte trafficking via the VCAM-1 and CCR2 axes, influences Annexin A1-dependent efferocytosis, and undergoes functional reprogramming through GRK2- and β-arrestin-mediated signaling. These mechanisms highlight the dynamic and context-dependent nature of immune β2-AR signaling, while also helping to explain why therapeutic modulation of this pathway has shown inconsistent clinical efficacy despite encouraging experimental findings. Current therapeutic strategies include selective β-blockade and GRK2-targeted interventions, whereas emerging approaches such as immune cell-specific β2-AR restoration, βARKct gene delivery, and biomaterial-based pro-resolving therapies remain at the preclinical stage. This review summarizes the expression patterns, signaling mechanisms, and immunoregulatory functions of β2-AR in immune cells, discusses its context-dependent roles across myocardial infarction, ischemia/reperfusion injury, and heart failure, and highlights current translational challenges and future opportunities for precision immunomodulation targeting immune cell β2-AR signaling.

## 1 Introduction

Cardiovascular disease and myocardial injury—including myocardial infarction (MI) and ischemia/reperfusion (I/R) injury—continue to impose a substantial global health burden and contribute to the development of heart failure (HF) despite advances in reperfusion strategies and guideline-directed pharmacotherapy (1, 2). Although contemporary treatments have significantly improved survival, a considerable proportion of patients still develop adverse ventricular remodeling and progressive heart failure, indicating that important pathogenic mechanisms remain insufficiently controlled (2). Accumulating evidence indicates that myocardial injury is not solely a consequence of cardiomyocyte loss but rather a dynamic process encompassing sterile inflammation, immune activation, tissue repair, and ventricular remodeling (2). Timely recruitment and coordinated activation of immune cells are essential for the clearance of necrotic tissue and initiation of repair; however, excessive or unresolved inflammation promotes fibrosis, adverse remodeling, and deterioration of cardiac function (2, 3). Consequently, understanding the mechanisms that govern immune responses after myocardial injury has become a major focus in cardiovascular research and offers new opportunities for therapeutic intervention.

Growing interest in immunocardiology has highlighted the intimate crosstalk between the sympathetic nervous system and the immune system during cardiovascular injury. Among adrenergic receptors, the β2-adrenergic receptor (β2-AR), a G protein-coupled receptor widely expressed on macrophages, neutrophils, dendritic cells, T lymphocytes, and other immune cell populations, has emerged as a key molecular link between neurohumoral activation and immune regulation (4, 5). Activation of immune cell β2-AR regulates leukocyte migration, polarization, cytokine production, phagocytosis, and efferocytosis through both canonical cAMP/PKA signaling and β-arrestin-dependent pathways, thereby shaping inflammatory initiation, resolution, and tissue repair (5). Importantly, accumulating evidence indicates that the biological effects of β2-AR are highly context dependent. Studies of acute myocardial injury and chronic adrenergic stress have reported distinct effects of immune-cell β2-AR signaling; however, these findings derive from different immune populations, genetic strategies, and experimental models and therefore do not establish a fixed temporal sequence of β2-AR function (6, 7). These observations suggest that immune-cell β2-AR functions as a dynamic regulator whose effects vary according to immune-cell subtype, experimental context, disease stage, and local microenvironment, extending far beyond its traditional role in cardiovascular physiology.

Despite rapid progress in elucidating immune cell β2-AR biology, several important challenges remain. Current evidence is largely derived from isolated experimental studies focusing on individual immune cell populations or specific signaling pathways, resulting in a fragmented understanding of how β2-AR coordinates immune responses throughout the different phases of myocardial injury. In this review, we integrate current knowledge regarding the expression patterns, signaling mechanisms, and immunoregulatory functions of β2-AR in immune cells, discuss its context-dependent roles in myocardial infarction, ischemia/reperfusion injury, and heart failure, and summarize emerging therapeutic strategies and translational challenges, with the aim of providing a comprehensive framework for precision immunomodulation targeting immune cell β2-AR signaling.

## 2 Expression and signaling mechanisms of β2-adrenergic receptor in immune cells

### 2.1 Expression profile and distribution characteristics

Early studies established β2-AR as the predominant adrenergic receptor subtype expressed in both innate and adaptive immune cells, providing early evidence that sympathetic signaling directly regulates immune function (4). Subsequently, *Thapa* and *Cao* (8) further proposed that β2-AR functions as a central neuroimmune signaling hub by coordinating immune-cell activation, migration, differentiation, and cytokine production to maintain immune homeostasis under physiological and pathological conditions. More recently, *Grisanti* et al. (6) demonstrated that leukocyte-specific β2-AR deficiency impaired immune-cell mobilization and recruitment to the injured myocardium, resulting in markedly increased post-myocardial infarction mortality, thereby providing direct evidence that immune-cell β2-AR is indispensable for coordinated inflammatory responses and cardiac repair following myocardial injury. These findings collectively established β2-AR as a key molecular interface linking sympathetic activation with immune regulation in cardiovascular disease.

The β2-AR is the predominant β-adrenergic receptor subtype expressed in immune cells, although its abundance varies substantially among different immune cell populations and differentiation states (4). Because available studies differ in species, activation state, and assay methodology, comparisons of β2-AR abundance across immune-cell populations should be interpreted qualitatively rather than as standardized quantitative rankings. Transcript abundance, receptor protein or surface density, ligand-binding capacity, and functional responsiveness represent related but distinct measures of receptor biology. Among circulating leukocytes, neutrophils express abundant functional β2-AR, although quantitative estimates vary across experimental settings. β2-AR is likewise the dominant adrenergic receptor subtype in monocytes and macrophages, whereas both CD4^+^ and CD8^+^ T lymphocytes express functional β2-AR, with comparatively lower expression reported in Th2 cells. Functional β2-AR has also been identified on B cells, natural killer (NK) cells, and dendritic cells, highlighting its broad distribution across both innate and adaptive immune compartments (8). Collectively, this widespread yet heterogeneous expression pattern positions β2-AR as an important interface linking sympathetic signaling to immune regulation. The expression profile of β2-AR across major immune cell subsets is summarized in **Table 1**.

**Table 1:** The expression profile of β2-AR across major immune cell subsets.

Immune cell subset	Relative β2-AR expression	Expression characteristics	Major β2-AR-regulated functions	Evidence considerations	Refs
Neutrophils	Predominant functional β2-AR; abundance estimates vary across studies	Functional β2-AR is well established in neutrophils, but quantitative receptor-abundance estimates vary according to assay, activation state, and species.	Chemotaxis and trafficking; adhesion; oxidative burst and degranulation; cytokine release; inflammatory resolution.	Evidence is strong for functional β2-AR signaling, but absolute receptor-density estimates vary with assay, activation state, and species.	(9, 10)
Monocytes	Predominant β-AR subtype; state-dependent	β2-AR is the dominant β-adrenergic receptor subtype in circulating monocytes, although expression changes during differentiation and activation.	TNF-α, IL-6, and IL-10 production; migration; Toll-like receptor responsiveness; differentiation into macrophages.	Most evidence derives from radioligand-binding studies, primary monocytes, and monocytic cell models.	(8, 9, 11)
Macrophages	Functional expression; state-dependent	β2-AR expression is generally higher after monocyte-to-macrophage differentiation and varies with polarization, tissue origin, and inflammatory context.	Inflammatory cytokine production; phagocytosis; polarization; tissue repair and resolution; metabolic reprogramming.	β2-AR effects are context dependent and may be anti- or pro-inflammatory according to timing, ligand concentration, and macrophage phenotype.	(12–15)
CD4^+^ T cells	Functional expression; subset-dependent	Functional β2-AR is present on naïve and activated CD4+ T cells; expression is dynamically regulated during differentiation.	Activation and proliferation; IL-2 and IFN-γ production; differentiation of Th1, Th17, and regulatory T-cell programs; metabolic reprogramming.	Direct comparison across subsets is complicated by activation-dependent and epigenetic regulation of ADRB2.	(16–19)
Th1 versus Th2 cells	Th1: detectable/high after activation; Th2: low or absent in classic models	Classic murine studies found β2-AR expression on activated Th1 cells but little or no detectable expression on activated Th2 cells.	Selective suppression of Th1-type cytokine responses; altered T-cell help for B cells; modulation of Th1/Th2 balance.	The Th1–Th2 contrast is best established in experimental differentiation systems and should not be generalized to every human tissue setting.	(17, 18)
CD8^+^ T cells	Functional expression; differentiation-dependent	Functional β2-AR is expressed by CD8+ T cells, with relatively high ADRB2 expression reported in differentiated effector-memory populations.	Cytotoxicity; IFN-γ production; trafficking; activation-induced metabolic reprogramming; antitumor immune responses.	Expression and functional sensitivity differ between naïve, central-memory, effector-memory, and exhausted states.	(20)
B cells	Predominant β-AR subtype; activation-dependent	B lymphocytes express almost exclusively β2-AR among β-adrenergic receptor subtypes; expression is regulated by activation and differentiation.	Antibody production; class switching; antigen presentation; cytokine secretion; interaction with helper T cells.	Functional effects depend strongly on the timing of receptor stimulation relative to B-cell receptor and CD40 signaling.	(17)
Natural killer cells	Functional expression; differentiation-dependent	Human NK cells express functional β2-AR; mature cytotoxic subsets may show particularly strong catecholamine responsiveness.	Rapid mobilization into blood; tissue trafficking; cytotoxicity; perforin and granzyme expression; IFN-γ production.	Acute β2-AR stimulation can mobilize NK cells, whereas sustained stimulation may suppress cytotoxic function.	(21, 22)
Dendritic cells	Functional expression; maturation-dependent	Functional β2-AR is present on conventional and monocyte-derived dendritic cells and on Langerhans cells; expression and signaling vary with maturation.	IL-10 and inflammatory cytokine production; migration; antigen presentation; cross-presentation; CD8+ T-cell priming.	Evidence supports robust functional signaling, but receptor abundance has not been quantified consistently across dendritic-cell subsets.	(8, 23)

Expression descriptors are qualitative summaries of findings reported within individual studies and should not be interpreted as standardized quantitative rankings across immune-cell populations. Available studies differ in species, activation state, and assay methodology. Transcript abundance, total receptor protein, cell-surface receptor density, ligand-binding capacity, and functional responsiveness represent related but distinct measures and are not considered interchangeable.

Unlike its relatively stable expression under physiological conditions, immune cell β2-AR is dynamically regulated by the inflammatory and neurohumoral microenvironment (8). Pro-inflammatory cytokines such as TNF-α and IL-1β attenuate receptor expression and signaling responsiveness, whereas glucocorticoids enhance β2-AR transcription and improve receptor coupling efficiency (24). Sustained sympathetic activation represents another major determinant of receptor regulation. In heart failure, prolonged exposure to elevated catecholamine levels promotes progressive β2-AR desensitization through PKA- and GRK-dependent phosphorylation, leading to receptor uncoupling, internalization, and reduced cell-surface receptor availability. The extent of receptor downregulation depends on both the duration and intensity of adrenergic stimulation as well as the cellular context (21, 25). Clinically, patients with chronic heart failure frequently exhibit persistently elevated plasma norepinephrine concentrations, often exceeding 600 pg/mL, which correlate with impaired β-adrenergic responsiveness and receptor downregulation (26, 27). Beyond compromising cardiomyocyte signaling, these neurohumoral alterations are increasingly recognized as potential modulators of immune cell β2-AR signaling, thereby influencing inflammatory activation, immune homeostasis, and ventricular remodeling.

### 2.2 Differential roles of β1-adrenergic receptor and β2-adrenergic receptor in cardiomyocytes and immune cells

Three β-adrenergic receptor subtypes have been identified: β1-AR, β2-AR, and β3-AR (28). This review focuses primarily on β1-AR and β2-AR because β1-AR predominates in cardiac adrenergic regulation, whereas β2-AR has been more extensively characterized in immune cells. β3-AR is expressed at relatively low levels in the normal myocardium but may be upregulated under pathological conditions and exhibits distinct Gi/NO-related signaling (26). However, direct evidence linking immune-cell β3-AR signaling to myocardial injury remains limited; therefore, β3-AR is not discussed in detail in this review. Understanding the differences between β1-AR and β2-AR provides an essential framework for interpreting the specific role of β2-AR signaling in cardiovascular immunity and inflammation. Historically, the field has evolved through several landmark contributions. *Xiao* and colleagues (29) demonstrated physiologically relevant coupling of β2-AR to Gi proteins in cardiomyocytes. Subsequently, *Padro* et al. (30) established the central immunomodulatory role of β2-AR in immune cells. More recently, *Chhatar and Lal* (5) further positioned β2-AR as a pivotal neuroimmune interface linking the sympathetic nervous system with the immune system. Under physiological conditions, β1-AR is the predominant adrenergic receptor subtype in cardiomyocytes, mediating most positive inotropic and chronotropic responses through the canonical Gαs–cAMP–PKA signaling pathway (31). Persistent β1-AR activation following myocardial injury promotes calcium dysregulation, maladaptive ventricular remodeling, and increased arrhythmogenic susceptibility (26, 32). In contrast, β2-AR constitutes a smaller proportion of myocardial β-adrenergic receptors but exhibits greater signaling versatility, including the ability to switch between Gαs- and Gαi-dependent signaling under pathological conditions (29, 31). *Lorton* et al. (33) first proposed that β2-AR-mediated immunoregulation exhibits remarkable signaling plasticity, with its biological effects being jointly determined by the duration of receptor activation, immune cell type, the local inflammatory microenvironment, and downstream signaling pathways. Subsequently, *Grisanti* et al. (6) demonstrated an essential role for leukocyte β2-AR in acute myocardial injury, whereas *Tanner* et al. (7)identified a distinct contribution of immune-cell β2-AR under chronic adrenergic stimulation.

In contrast to cardiomyocytes, β2-AR is the predominant β-adrenergic receptor subtype expressed in immune cells, whereas β1-AR expression is relatively low and contributes minimally to adrenergic regulation in most immune cell populations (8, 38). This differential receptor distribution underlies the dominant role of β2-AR in controlling immune cell migration, polarization, cytokine production, and inflammatory resolution. Importantly, β2-AR signaling in immune cells is highly dynamic, with receptor expression and signaling responsiveness being continuously shaped by immune activation status and the local inflammatory microenvironment. The distinct cellular distribution of β1-AR and β2-AR has important therapeutic implications. Non-selective β-blockers inhibit both receptor subtypes and therefore simultaneously suppress myocardial β1-AR signaling and immune cell β2-AR activity (39). By contrast, β1-selective β-blockers preferentially target cardiomyocyte β1-AR while largely preserving immune β2-AR signaling at conventional therapeutic doses, although this selectivity diminishes at higher drug concentrations (36). These observations suggest that preserving or selectively modulating immune cell β2-AR signaling may provide a more precise strategy to regulate post-infarction inflammation while minimizing interference with β1-AR-mediated cardiac contractile and electrophysiological functions. The major differences in β1-AR and β2-AR signaling between cardiomyocytes and immune cells are summarized in **Table 2**.

**Table 2:** Comparison of β1-AR and β2-AR signaling in cardiomyocytes and immune cells.

Feature	β1-AR in cardiomyocytes	β2-AR in cardiomyocytes	β2-AR in immune cells	Refs
Dominant expression	Predominant at the whole-myocardium/tissue level (~70–80% of β-ARs); broadly detected in ventricular myocytes	Lower whole-myocardium/tissue proportion (~20–30% of β-ARs); detected in only a subset of individual ventricular myocytes	Predominant β-AR subtype	(8, 31)
G protein coupling	Gs	Gs and Gi	Primarily Gs with context-dependent Gi	(8, 34)
cAMP signaling	Global/diffuse	Compartmentalized	Compartmentalized	(28, 35)
Major downstream pathways	PKA, CaMKII	PKA, PI3K-Akt, ERK, β-arrestin	PKA, CREB, EPAC, β-arrestin	(28, 35)
Primary biological function	Excitation–contraction coupling	Cardioprotection & fine tuning	Immunoregulation	(8, 28)
Effect of chronic activation	Hypertrophy, apoptosis, HF progression	Relative cytoprotection (context-dependent)	Context-dependent immune modulation	(21, 26)
Representative therapeutic implication	β1-selective blockers	β2-biased agonism (experimental)	Immune-targeted β2 modulation	(6, 36, 37)

### 2.3 β1- and β2-adrenergic receptor autoantibodies

Beyond endogenous catecholamine signaling, functional autoantibodies against β-adrenergic receptors provide an additional mechanism capable of modifying adrenergic receptor activity in cardiovascular disease. β1-AR autoantibodies (β1-AA) are the best characterized, particularly in dilated cardiomyopathy and heart failure, where agonistic antibodies can sustain β1-AR signaling, promote receptor desensitization, and contribute to myocardial dysfunction (40, 41). β2-AR autoantibodies (β2-AA) have also been identified in patients with cardiovascular disease, although their functional effects appear more heterogeneous; experimental studies suggest that β2-AA may modify β1-AA-associated myocardial injury (42). Therapeutic approaches including immunoadsorption and antibody-neutralizing strategies have been investigated, particularly in β1-AA-positive cardiomyopathy, but their broader clinical utility remains uncertain (41). Whether β1-AA or β2-AA directly influences immune-cell adrenergic signaling during myocardial injury remains to be established.

### 2.4 Diversity and regulation of signaling pathways

Unlike many G protein-coupled receptors that signal predominantly through a single downstream pathway, β2-AR exhibits remarkable signaling versatility. Depending on receptor activation status, phosphorylation pattern, and cellular context, β2-AR can engage distinct signaling modules that collectively determine immune-cell behavior and inflammatory outcomes. These signaling mechanisms are summarized in **Figure 1**.

**Figure 1:**
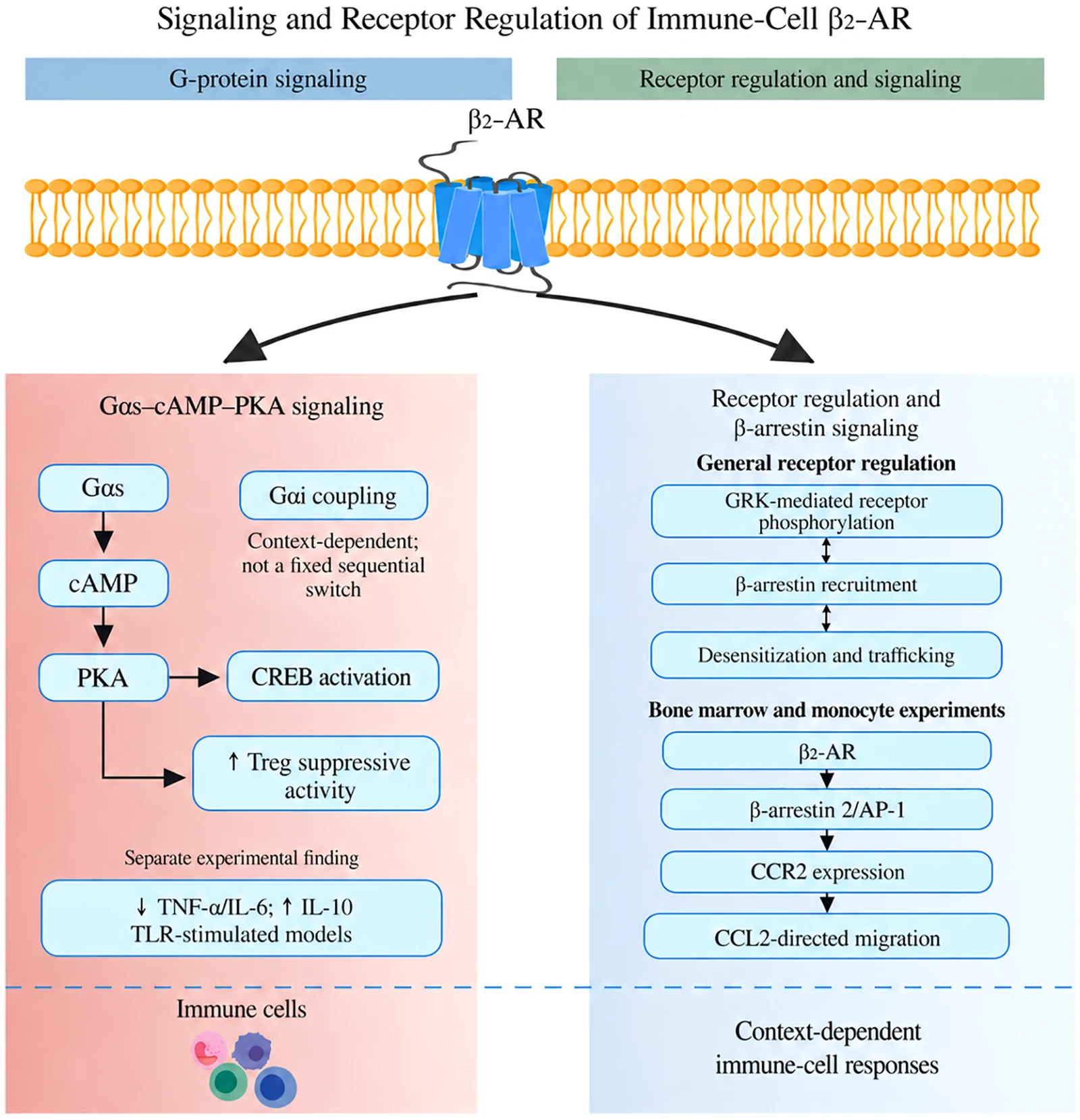
Signaling and receptor regulation of immune-cell β2-AR. β2-AR engages G-protein signaling and GRK/β-arrestin-mediated receptor regulation. Gαs–cAMP–PKA signaling supports CREB activation and Treg suppressive activity, while β-arrestin 2/AP-1 regulates CCR2 expression and CCL2-directed migration in bone marrow cells and monocytes. The cytokine changes shown derive from TLR-stimulated models, and G-protein coupling is context dependent.

Under physiological conditions, β2-AR primarily couples to the stimulatory Gαs, initiating the canonical cAMP–PKA signaling cascade. Activation of adenylate cyclase increases intracellular cAMP levels, leading to PKA activation and subsequent phosphorylation of downstream substrates, including the transcription factor CREB. Through inhibition of NF-κB activity and suppression of pro-inflammatory cytokine production, such as TNF-α and IL-6, the Gαs–cAMP–PKA pathway constitutes the principal mechanism underlying the anti-inflammatory effects of β2-AR signaling (35). However, β2-AR signaling is not static. In addition to canonical Gαs coupling, β2-AR can engage Gαi in a context-dependent manner (29, 34, 43). In some experimental systems, PKA-dependent receptor phosphorylation can modify G-protein coupling preferences (44). Separately, GRK-mediated receptor phosphorylation and β-arrestin recruitment contribute to receptor desensitization, trafficking, and signaling reprogramming (45–47). These distinct signaling modes may differentially influence downstream pathways according to receptor state and cellular context.

Increasing attention has recently focused on β-arrestin-dependent signaling, which is now recognized as an independent signaling platform rather than merely a mechanism of receptor desensitization. Beyond mediating receptor internalization, β-arrestins function as scaffold proteins that coordinate multiple downstream pathways, including ERK1/2, MAPK, and EGFR signaling. β2-AR-dependent regulation of CCR2 expression through the β-arrestin 2/AP-1 axis further highlights its role in controlling monocyte migration and inflammatory cell trafficking (11). Accumulating evidence indicates that the two β-arrestin isoforms fulfill distinct biological functions. In cardiomyocytes, β-arrestin 2 preferentially activates pro-survival pathways, including ERK1/2 and Akt, thereby limiting apoptosis and improving myocardial resilience after injury. In contrast, β-arrestin 1 has more frequently been associated with sustained GRK2 activation, impaired receptor responsiveness, and adverse ventricular remodeling (47, 48). Similar isoform-specific divergence has also been reported in immune cells, where β-arrestin 2 promotes CCR2-dependent leukocyte migration (11) and anti-inflammatory signaling (49), whereas β-arrestin 1 may facilitate NF-κB activation and amplify inflammatory responses under certain pathological conditions (50). These observations indicate that β-arrestin signaling is determined by isoform engagement, cell type, and disease context rather than representing a uniform downstream response.

Recognition of this signaling diversity has important therapeutic implications. Instead of globally enhancing or inhibiting β2-AR activity, selectively modulating individual signaling branches has emerged as a more attractive strategy. In particular, biased ligands capable of preferentially promoting β-arrestin 2-dependent protective signaling while minimizing maladaptive G protein- or β-arrestin 1-mediated responses have attracted increasing interest as next-generation therapeutic agents (51). Such approaches may enable more precise regulation of immune and cardiac responses following myocardial injury, although their clinical application will require careful consideration of disease context, tissue specificity, and receptor signaling status.

## 3 Immune cell β2-adrenergic receptor signaling in myocardial injury

Myocardial injury triggers a coordinated immune response involving both circulating leukocytes recruited from peripheral reservoirs and immune populations already residing within the cardiac tissue. These two compartments differ in cellular origin, recruitment dynamics, and functional roles during injury and repair, and should therefore not be considered interchangeable. Current evidence indicates that immune-cell β2-AR signaling participates in several key processes relevant to myocardial injury, including leukocyte mobilization, chemokine-dependent recruitment, inflammatory regulation, and efferocytosis. However, the strength of evidence differs substantially between circulating/recruited and cardiac-resident immune populations. Most direct mechanistic studies have focused on hematopoietic, bone marrow-derived, or recruited immune cells, whereas the cell-autonomous role of β2-AR in cardiac-resident immune populations remains less well defined.

### 3.1 Circulating and recruited immune cells

Circulating and recruited leukocytes constitute a major component of the inflammatory response to myocardial injury. Following myocardial infarction, immune cells are mobilized from peripheral reservoirs, including the spleen and bone marrow, and subsequently recruited to the injured myocardium in response to adhesion molecules and chemokine gradients. Within this process, immune-cell β2-AR signaling has been directly linked to both splenic leukocyte retention and CCR2-dependent chemotaxis, providing some of the strongest mechanistic evidence connecting adrenergic signaling to immune-cell trafficking after myocardial injury (6, 11, 52–55).

#### 3.1.1 Splenic retention and the regulatory role of VCAM-1

A major advance in understanding immune cell β2-AR function came from the discovery that β2-AR signaling governs leukocyte trafficking through regulation of splenic immune-cell retention. *Swirski* et al. (52) first demonstrated that the spleen is not merely a passive reservoir of leukocytes but serves as a major reservoir of inflammatory monocytes following myocardial infarction. Upon myocardial injury, large numbers of splenic monocytes are rapidly mobilized into the circulation and recruited to the infarcted myocardium, where they contribute to the clearance of necrotic tissue and initiation of early cardiac repair. Building upon this concept, *Dutta* et al. (53) further revealed that the spleen not only stores mature immune cells but also supports extramedullary myelopoiesis by retaining hematopoietic stem and progenitor cells through VCAM-1-dependent adhesion. *Grisanti* et al. (6) subsequently demonstrated that deletion of β2-AR in immune cells markedly increased splenic VCAM-1 expression, resulting in abnormal retention of monocytes, neutrophils, and mast cells within the spleen and reduced recruitment of these cells to the infarcted myocardium. These findings established the β2-AR–VCAM-1 axis as a key mechanism regulating the mobilization of splenic immune cells after myocardial infarction. More recently, *Akbar* et al. (54) showed that the injured myocardium communicates remotely with the spleen through endothelial cell-derived extracellular vesicles carrying VCAM-1 and miR-126, thereby inducing splenic inflammatory gene expression and rapidly mobilizing splenic neutrophils within hours after myocardial infarction. These observations are consistent with the findings of *Gladow* et al. (55), who demonstrated that CD4^+^ T cells actively regulate splenic myelopoiesis after myocardial infarction, with conventional CD4^+^ T cells promoting myeloid cell expansion and inflammatory monocyte differentiation, whereas regulatory T cells exert inhibitory effects on this process.

Collectively, these studies redefine the spleen as an active immunological hub that orchestrates inflammatory cell mobilization, emergency myelopoiesis, and tissue repair following myocardial infarction. Importantly, this regulatory mechanism is not restricted to experimental models. Patients receiving metoprolol exhibit increased splenic VCAM-1 expression, consistent with observations in β2-AR-deficient mice. Furthermore, splenectomy partially restored cardiac leukocyte recruitment in hematopoietic β2-AR knockout mice, supporting an important contribution of splenic leukocyte retention to impaired recruitment after myocardial injury (6). Collectively, these findings identify the β2-AR–VCAM-1 axis as a central mechanism coordinating immune-cell release from the splenic reservoir and subsequent trafficking to injured cardiac tissue (**Figure 2**).

**Figure 2:**
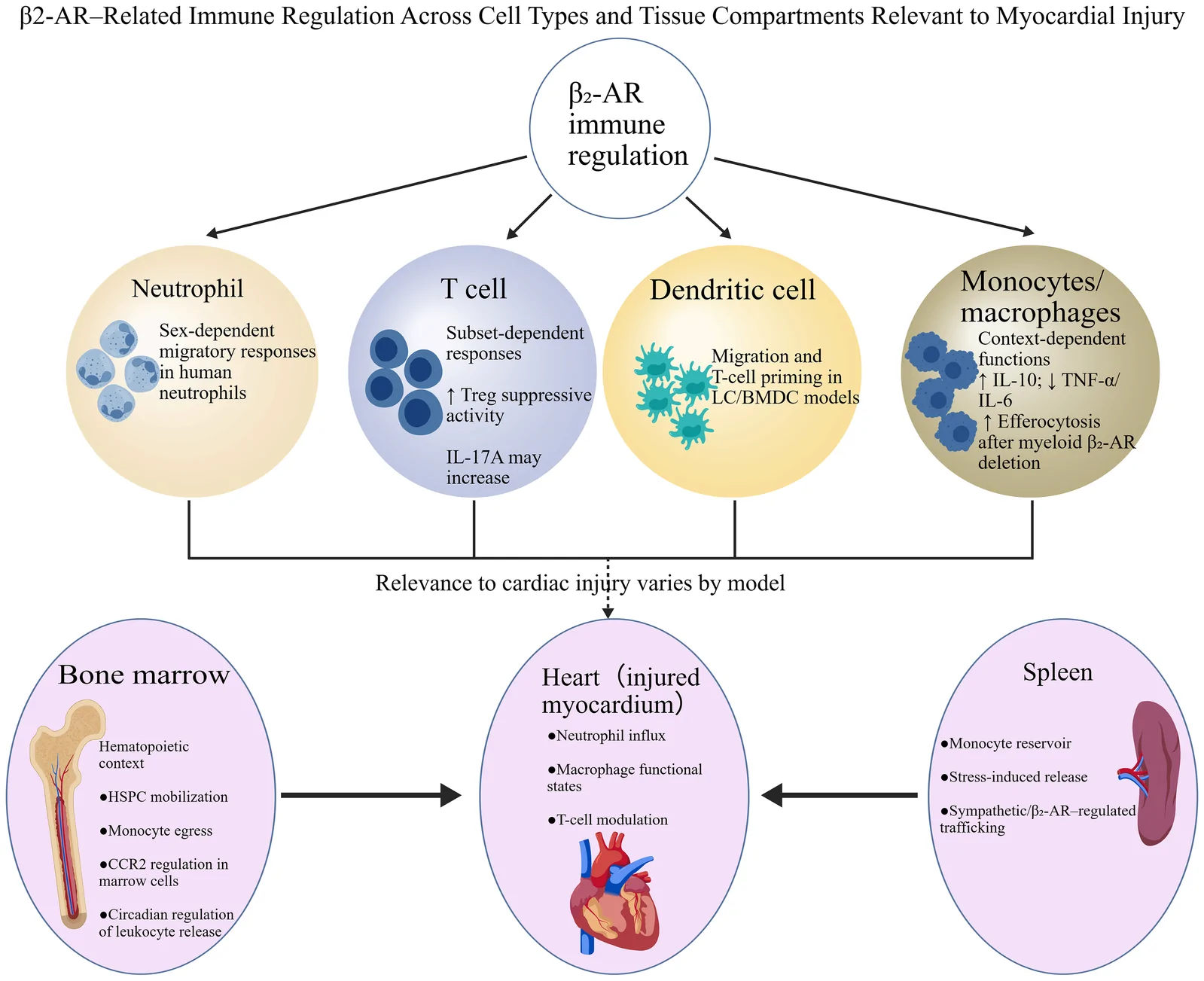
β2-AR-related immune regulation across cell types and tissue compartments relevant to myocardial injury. The schematic summarizes β2-AR-related responses in neutrophils, T cells, dendritic cells, and monocytes/macrophages, together with leukocyte trafficking among the bone marrow, spleen, and injured myocardium. These findings derive from different experimental systems, and their relevance to myocardial injury varies by cell type and model. Arrows between tissue compartments indicate leukocyte trafficking.

#### 3.1.2 CCR2-dependent leukocyte recruitment and chemotaxis

The β2-AR plays a significant role in modulating the migratory capacity of monocytes and macrophages by regulating the expression and function of the chemokine receptor CCR2 (8). Research has shown that β2-AR upregulates CCR2 expression through the β-arrestin 2/AP-1 signaling pathway, thereby enhancing monocyte chemotaxis toward CCL2 (MCP-1) (11). In contrast, genetic deletion of β2-AR abolished CCR2 expression in bone marrow-derived monocytes and markedly impaired their migratory response to CCL2, whereas reconstitution of β2-AR restored both receptor expression and chemotactic capacity. Subsequent studies further established CCR2 as a critical downstream effector of immune-cell β2-AR signaling. Mice lacking leukocyte β2-AR exhibited markedly reduced recruitment of monocytes/macrophages and neutrophils to the infarcted myocardium after myocardial infarction. Notably, a nearly identical phenotype was observed in chimeric mice with immune cell-specific CCR2 deficiency and in wild-type mice treated with pharmacological CCR2 antagonists, indicating that impaired leukocyte recruitment following β2-AR deletion is largely mediated through disruption of CCR2-dependent chemotaxis. Conversely, pharmacological activation of β2-AR increased bone marrow CCR2 expression and enhanced leukocyte migration toward CCL2 gradients.

Collectively, these findings identify the β2-AR-CCR2 axis as a key regulatory pathway governing inflammatory-cell mobilization after myocardial infarction. Rather than directly controlling leukocyte migration, β2-AR primarily regulates the sensitivity of immune cells to chemokine gradients by maintaining CCR2 expression, thereby ensuring efficient recruitment of reparative leukocytes to injured cardiac tissue.

### 3.2 Cardiac-resident immune cells

In parallel with the recruitment of circulating leukocytes, the myocardium contains heterogeneous resident immune populations that contribute to immune surveillance, cardiac homeostasis, and tissue responses following injury. Among these populations, cardiac-resident macrophages have been most extensively characterized. Single-cell and lineage-based studies have demonstrated that resident cardiac macrophages are highly heterogeneous and differ from recruited monocyte-derived macrophages in developmental origin, localization, transcriptional state, and functional responses to myocardial injury (56–59). Notably, self-renewing resident macrophages have been shown to limit adverse ventricular remodeling after myocardial infarction, whereas distinct resident CCR2− and CCR2+ macrophage populations differentially regulate monocyte recruitment and fate specification following cardiac injury (57, 59). These differences are particularly important when interpreting the role of β2-AR signaling in myocardial injury. Much of the available mechanistic evidence has been derived from global hematopoietic deletion, bone marrow transplantation, myeloid-restricted deletion, or bone marrow-derived immune-cell models (6, 7, 11, 60). Although these studies establish an important role for immune-cell β2-AR signaling, they do not fully distinguish the relative contributions of circulating/recruited immune cells and pre-existing cardiac-resident populations. Accordingly, findings obtained from bone marrow-derived or recruited immune cells cannot necessarily be directly extrapolated to cardiac-resident immune populations.

Importantly, direct evidence defining β2-AR signaling specifically within cardiac-resident macrophages or other resident immune-cell subsets remains limited. This knowledge gap is particularly relevant because resident and recruited macrophage populations can perform distinct and complementary functions during myocardial injury. Further studies using lineage-resolved and inducible genetic approaches will therefore be necessary to determine whether β2-AR signaling differentially regulates these immune compartments and how their interactions influence cardiac inflammation and repair.

### 3.3 Regulation of annexin A1 and efferocytosis

Beyond regulating leukocyte recruitment, β2-AR signaling also participates in the resolution phase of post-infarction inflammation by modulating macrophage efferocytosis. Efficient clearance of apoptotic cells is essential for terminating inflammatory responses, limiting secondary tissue injury, and promoting cardiac repair. Nayak et al. (60) recently demonstrated that myeloid cell-specific deletion of β2-AR markedly increased Annexin A1 (Anxa1) expression in bone marrow-derived myeloid cells and cardiac CD11b+ cells following myocardial infarction. Annexin A1 is a well-established pro-resolving mediator that facilitates recognition and engulfment of apoptotic cells through activation of the FPR2/ALX receptor on macrophages (61). Consistent with increased Anxa1 expression, β2-AR-deficient bone marrow-derived macrophages displayed significantly enhanced efferocytosis *in vitro*, whereas shRNA-mediated Anxa1 knockdown reduced this enhanced efferocytic phenotype (60). Together, these findings indicate that loss of β2-AR signaling in myeloid cells enhances Anxa1-dependent efferocytosis, accompanied by accelerated neutrophil clearance and improved cardiac function and remodeling after myocardial infarction (60).

These findings differ from those obtained in the broader hematopoietic β2-AR deletion model reported by Grisanti et al. (6), in which loss of β2-AR impaired leukocyte recruitment to the injured myocardium and was associated with adverse cardiac outcomes. Rather than representing successive stages of the same biological process, the divergent phenotypes observed in these studies likely reflect differences in the immune-cell populations targeted and the genetic deletion strategies employed. These findings are consistent with the pioneering work of *Perretti* et al. (61), who established Annexin A1 as one of the principal endogenous pro-resolving mediators. During acute inflammation and tissue injury, Anxa1 is typically induced to limit neutrophil recruitment, promote macrophage-mediated efferocytosis, and restore tissue homeostasis. Building on this concept, *Gobbetti* (62) further proposed that increased Anxa1 expression should be regarded as a hallmark of activation of endogenous resolution programs following tissue injury, rather than merely an isolated anti-inflammatory event.

## 4 Immunomodulation of immune cell polarization and inflammatory response by β2-adrenergic receptor

### 4.1 Fine-tuned regulation of macrophage polarization

Macrophages are central orchestrators of inflammation, tissue repair, and ventricular remodeling following myocardial injury. As discussed above, the cardiac macrophage compartment comprises both resident and recruited populations with distinct ontogenies and responses to injury (56–59). Within these populations, macrophage activation is better understood as a continuum of functional states rather than a binary M1/M2 classification (63–65). In this context, β2-AR signaling should be viewed not as a simple inducer of anti-inflammatory macrophages but as a context-dependent regulator of macrophage functional plasticity (6, 7, 11).

Under acute inflammatory conditions, β2-AR signaling can modulate the activation of infiltrating inflammatory macrophages. Activation of the canonical Gαs–cAMP–PKA pathway suppresses NF-κB- and AP-1-dependent transcription, thereby reducing the production of IL-1β, TNF-α, iNOS, and other inflammatory mediators while promoting expression of anti-inflammatory molecules including IL-10, Arg-1, and TGF-β (13, 14, 35). In a chronic isoproterenol model using wild-type or β2-AR-deficient bone marrow chimeric mice, Tanner et al. (7) found that loss of immune-cell β2-AR reduced the cardiac accumulation of CCR2high pro-inflammatory macrophages, whereas CX3CR1+ macrophages were comparatively less affected. Cardiac macrophages from β2-AR-deficient bone marrow recipients also showed reduced expression of several inflammatory mediators under chronic adrenergic stimulation. These findings indicate that, in the setting of sustained catecholamine exposure, immune-cell β2-AR can facilitate the recruitment and inflammatory activity of pro-inflammatory macrophage populations.

During inflammation resolution, macrophage pro-resolving functions are regulated by multiple signaling pathways. cAMP–PKA signaling promotes macrophage reprogramming and efferocytosis, accompanied by increased AnxA1 and CD36 expression (15). In the ischemic heart, Sectm1a-mediated efferocytosis independently contributes to the clearance of apoptotic cells and limits persistent inflammation and cardiac injury (66). In parallel, Nayak et al. (60) demonstrated that myeloid-specific β2-AR deletion enhances Anxa1-dependent efferocytosis and improves post-infarction cardiac function and remodeling. Together, these studies support an important role for macrophage efferocytosis in inflammation resolution while indicating that the underlying regulatory mechanisms are context dependent rather than attributable to a single β2-AR-driven pathway. Rather than representing a distinct polarization program, these coordinated responses reflect progressive functional adaptation that enables macrophages to terminate inflammation while promoting structural repair of the injured myocardium. However, persistent sympathetic activation during chronic remodeling alters this regulatory program. Sustained catecholamine exposure induces β2-AR phosphorylation by GRKs, receptor desensitization, and β-arrestin-dependent signaling reprogramming, thereby attenuating canonical cAMP-mediated signaling response (26, 48). Consequently, prolonged β2-AR dysfunction may contribute to persistent inflammation, defective resolution, and adverse ventricular remodeling. Collectively, current evidence supports a model in which β2-AR acts as a dynamic regulator of macrophage functional plasticity rather than a simple promoter of M2 polarization. Its biological effects are determined by macrophage origin, activation state, and disease stage, highlighting the importance of temporally and cell-specific modulation of β2-AR signaling for optimizing post-infarction immune repair.

### 4.2 Multifaceted regulation of T cell function

Compared with its effects on innate immune cells, β2-AR signaling also exerts immunomodulatory effects within the adaptive immune system. Rather than globally suppressing T cell immunity, β2-AR signaling can differentially regulate T cell activation and cytokine production according to T-cell subset and experimental context (17–20). β2-AR/cAMP signaling has been associated with reduced proliferation and suppression of Th1-associated cytokines, including IL-2 and IFN-γ, in selected experimental settings (17, 18). However, its effects on Th17 responses are not uniformly suppressive. In human T cells, the β2-AR agonist terbutaline increased IL-17A production while reducing IFN-γ, indicating that β2-AR-dependent regulation of Th17 responses is context dependent (19). Regulatory T cells (Tregs) accumulate within the infarcted myocardium during the reparative phase and play indispensable roles in terminating inflammation, regulating fibroblast activation, and facilitating scar maturation (67–70). Separately, Guereschi et al. (16) demonstrated that CD4+Foxp3+ Tregs express functional β2-AR and that receptor activation increases intracellular cAMP and PKA-dependent CREB phosphorylation, enhances their suppressive activity, and increases CTLA-4 expression. These findings establish a direct effect of β2-AR on Treg suppressive function, although its contribution to Treg-mediated cardiac repair after myocardial infarction remains to be directly determined. Collectively, these findings indicate that β2-AR signaling modulates adaptive immune responses in a T-cell subset- and context-dependent manner, with distinct effects on conventional effector T cells and regulatory T-cell function. However, the extent to which these mechanisms directly shape T-cell trafficking and Treg-mediated repair in the injured myocardium remains to be established.

### 4.3 Complex modulation of neutrophil function

Neutrophils are among the earliest immune cells recruited to the injured myocardium and play indispensable roles in host defense, clearance of cellular debris, and initiation of tissue repair. However, excessive neutrophil activation amplifies myocardial injury through the release of reactive oxygen species (ROS), proteolytic enzymes, and pro-inflammatory mediators, making precise regulation of neutrophil responses essential for balancing tissue repair and inflammatory damage (71). Rather than simply suppressing neutrophil recruitment, β2-AR signaling can modulate neutrophil activation and inflammatory potential. Direct studies in neutrophils indicate that β2-AR/cAMP signaling can attenuate oxidative responses and regulate other functional properties (10, 72). Evidence for a β2-AR-specific role in neutrophil trafficking during myocardial injury, however, remains more limited. β-Arrestin 2-dependent regulation of CXCR2 signaling has been shown to influence neutrophil chemotaxis and receptor responsiveness (73), but this finding does not by itself establish direct involvement of β2-AR. These effects are highly dependent on the magnitude and duration of adrenergic stimulation, receptor desensitization status, and the surrounding inflammatory microenvironment, indicating that β2-AR modulates neutrophil function in a context-dependent manner rather than acting as a simple on–off switch.

A mechanistically distinct example of adrenergic regulation of neutrophils is the “neutrophil stunning” phenomenon described by García-Prieto et al. (74). In this study, early metoprolol administration during myocardial ischemia/reperfusion acted through β1-AR in the hematopoietic compartment and altered neutrophil behavior, reducing migration and tissue infiltration while disrupting neutrophil–platelet interactions. These effects contributed to reduced reperfusion injury but should not be interpreted as direct evidence for a β2-AR activation mechanism. Subsequent work further showed that this neutrophil-modulating effect was not shared uniformly across β-blockers, as metoprolol, but not atenolol or propranolol, reduced neutrophil migration and infiltration under the experimental conditions studied (71).

Collectively, current evidence indicates that β2-AR can regulate several aspects of neutrophil functional activity, whereas its specific contribution to neutrophil recruitment during myocardial injury remains incompletely defined. More broadly, the metoprolol studies demonstrate that adrenergic receptors can directly influence neutrophil dynamics during myocardial ischemia/reperfusion while also emphasizing the importance of receptor subtype and drug-specific mechanisms. Such fine-tuning may be particularly important during the early phase of myocardial injury, when excessive neutrophil activation contributes substantially to infarct expansion and adverse ventricular remodeling (**Figure 3**).

**Figure 3:**
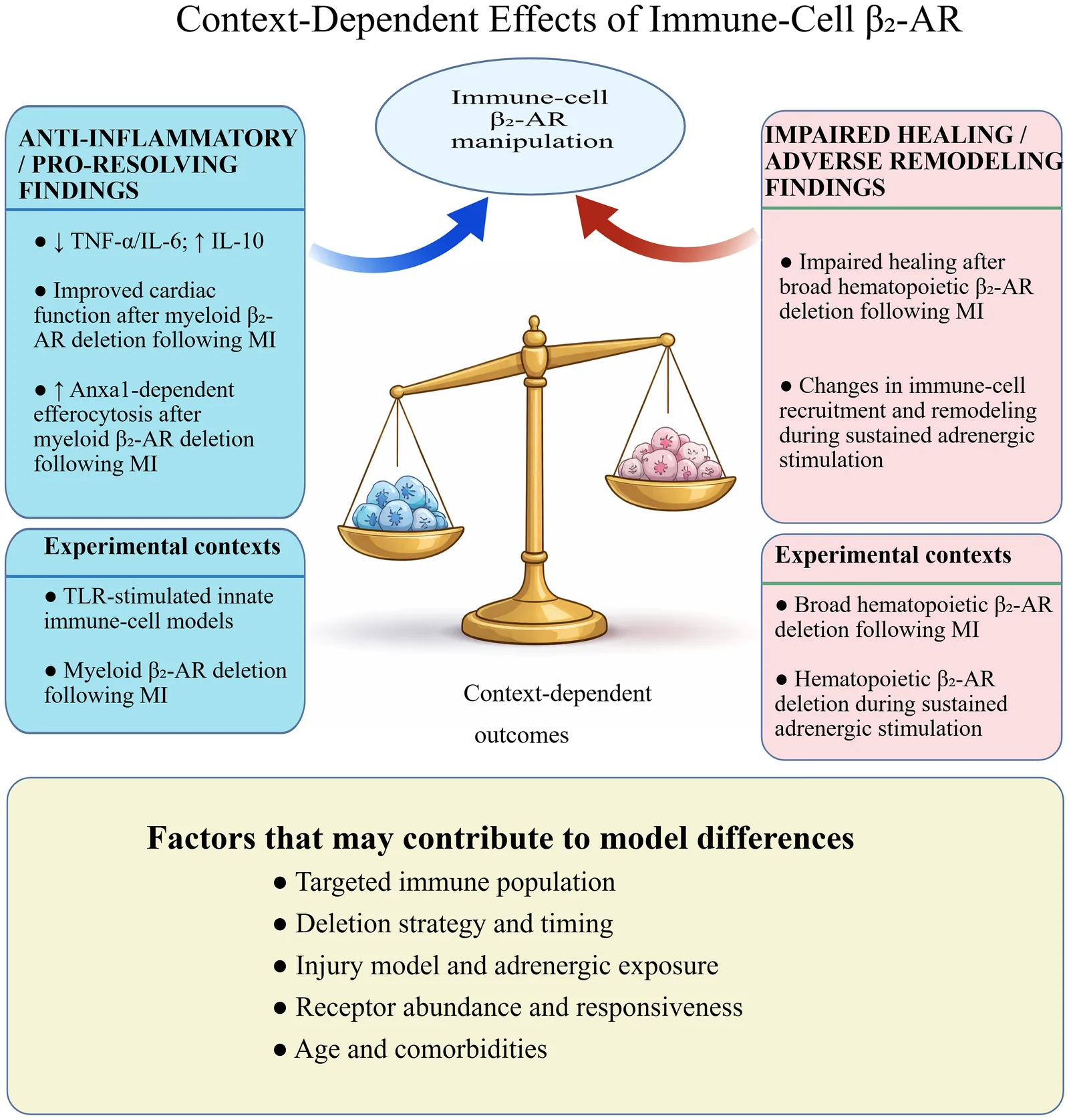
Context-dependent effects of immune-cell β2-AR manipulation. Myeloid-specific β2-AR deletion enhances Anxa1-dependent efferocytosis and improves cardiac function after MI, whereas broad hematopoietic deletion impairs post-infarction healing. Findings from TLR-stimulated models and sustained adrenergic stimulation provide additional experimental contexts. Differences in targeted cells, deletion strategies, injury models, and adrenergic exposure may contribute to divergent outcomes.

## 5 Therapeutic perspectives: strategies targeting immune cell β2-adrenergic receptor

### 5.1 The paradoxical effects and precision application of beta-blockers

β-blockers remain a cornerstone of pharmacological therapy for myocardial infarction and chronic heart failure. However, increasing evidence suggests that their therapeutic benefits extend beyond reduction of myocardial oxygen demand and neurohumoral activation. By modulating β2-adrenergic receptor (β2-AR) signaling in immune cells, β-blockers may also influence post-infarction inflammation and tissue repair. Consequently, the clinical effects of β-blockade depend not only on receptor inhibition itself but also on treatment timing, receptor selectivity, patient characteristics, and the evolving immunological environment. Current therapeutic strategies targeting immune-cell β2-AR signaling are summarized in **Table 3**.

**Table 3:** Current therapeutic strategies targeting immune-cell β2-AR signaling.

Therapeutic strategy	Representative agents	Primary target/mechanism	Effects on immune cells	Potential cardiovascular benefit	Evidence level
Non-selective β-blockers	Propranolol	β1/β2 blockade	Modulate inflammatory-cell recruitment and cytokine production in experimental settings	Established cardiovascular effects in selected indications; contribution of immune β2-AR mechanisms remains uncertain	Clinical cardiovascular evidence + preclinical immune-mechanistic evidence
β1-selective blockers	Metoprolol; Bisoprolol	Predominantly β1 blockade	Predominantly β1-mediated effects; preservation of immune β2-AR signaling is indirect and remains a proposed mechanism	Established benefit in MI/HF	Clinical cardiovascular evidence; immune β2-AR mechanism not established
β1/β2 + α1 blocker	Carvedilol	β1/β2/α1 blockade	Suppress inflammatory activation; affect macrophage polarization in experimental models	Improves ventricular remodeling	Clinical cardiovascular evidence + experimental immune-mechanistic evidence
β2-AR agonists	Salbutamol; Formoterol	β2-AR activation	Context-dependent anti-inflammatory signaling	Potential context-dependent effects; therapeutic efficacy in myocardial injury is not established	Predominantly preclinical mechanistic evidence
β2-AR–CCR2 axis modulation	Experimental β2 agonists; CCR2 antagonists	β2-AR–CCR2 pathway	Regulates monocyte/macrophage chemotaxis	Proposed modulation of leukocyte recruitment; therapeutic benefit after MI remains unestablished	Preclinical mechanistic evidence/proposed therapeutic application
Pro-resolving pathway modulation	Annexin A1-based strategies	Annexin A1-associated pro-resolving pathways	Promotes efferocytosis and inflammation resolution	Potential enhancement of cardiac repair; not established as a β2-AR-directed therapy	Preclinical pro-resolving evidence/proposed therapeutic application

Established cardiovascular benefits of clinically used adrenergic drugs should not be interpreted as evidence that these benefits are mediated by immune-cell β2-AR signaling. Likewise, preclinical mechanistic findings and experimental rescue studies establish biological plausibility or causality within specific models but do not by themselves demonstrate therapeutic efficacy in receptor-intact disease or clinical populations.

Clinical studies have clearly demonstrated that the timing of β-blocker initiation is a major determinant of therapeutic outcome. In the COMMIT/CCS-2 trial, immediate intravenous followed by oral metoprolol reduced reinfarction and ventricular fibrillation but increased the incidence of cardiogenic shock in hemodynamically unstable patients, indicating that indiscriminate early β-blockade may be harmful despite potential anti-inflammatory benefits (75). In contrast, the METOCARD-CNIC trial showed that carefully selected patients with anterior STEMI receiving intravenous metoprolol before reperfusion experienced smaller infarct size (76). Subsequent meta-analyses have likewise reported heterogeneous results, suggesting that the balance between benefit and risk is strongly influenced by infarct characteristics, reperfusion status, and baseline hemodynamic stability (77, 78). These clinical findings establish that the effects of β-blockade are strongly influenced by treatment timing and clinical context. However, these trials did not directly assess immune-cell β2-AR signaling, and their differing outcomes therefore cannot currently be attributed to stage-dependent immune mechanisms. Experimental studies raise the possibility that immune-cell β2-AR responses may modify the effects of adrenergic interventions, but this remains a mechanistic hypothesis that requires direct clinical validation. Future studies integrating immune phenotyping with receptor-specific functional measurements will be needed to determine whether immune-cell β2-AR status contributes to treatment response. These observations support a shift from the traditional concept of uniform β-blocker therapy toward precision β-adrenergic modulation. Future therapeutic strategies may include individualized selection of β1-selective versus non-selective β-blockers, optimization of treatment according to established clinical characteristics, and investigation of immune cell-targeted approaches that preserve or selectively modulate β2-AR signaling. Whether such immune-directed strategies provide additional clinical benefit remains to be determined.

### 5.2 Therapeutic potential and challenges of β2-adrenergic receptor agonists

β2-AR agonists have long been considered attractive therapeutic candidates because of their potent anti-inflammatory and immunomodulatory properties. However, despite encouraging experimental findings, their clinical application in myocardial injury has remained limited. This discrepancy largely reflects the narrow therapeutic window of conventional β2-AR agonists and the complex, context-dependent nature of β2-AR signaling during cardiac injury and repair (25, 37). Experimental studies indicate that β2-AR activation can modulate inflammatory signaling and cytokine production under selected conditions; however, these findings do not establish a clinically validated therapeutic window for β2-AR agonism after myocardial injury. Nevertheless, direct clinical evidence supporting β2-AR agonists in acute myocardial infarction remains scarce. Existing cardiovascular trials have primarily evaluated physiological or hemodynamic outcomes rather than immune modulation. Small randomized studies using β2-AR agonists such as albuterol and clenbuterol demonstrated improved pulmonary vascular reserve with albuterol (79) and increased lean body mass with clenbuterol (80) in selected heart failure populations but were not designed to assess post-infarction immune responses. However, endurance and exercise duration decreased after clenbuterol treatment (80).

Despite these promising short-term effects, prolonged β2-AR stimulation presents substantial therapeutic challenges. Sustained agonist exposure promotes receptor phosphorylation by GRKs, recruitment of β-arrestins, and progressive receptor desensitization, resulting in impaired canonical cAMP signaling and diminished biological responsiveness (25, 45, 46). Furthermore, adverse cardiovascular effects—including tachycardia, arrhythmias, and hypokalemia—have further restricted chronic use of conventional β2-AR agonists in patients with myocardial injury. These limitations indicate that simply increasing β2-AR activation is unlikely to provide sustained therapeutic benefit.

Recognition of these challenges has shifted attention toward biased β2-AR agonists, which aim to preferentially engage selected downstream signaling pathways while minimizing receptor desensitization and off-target effects. Experimental pharmacological studies have identified several ligands capable of preferentially activating distinct β2-AR signaling programs. For example, isoetharine exhibits relatively enhanced β-arrestin-associated signaling compared with canonical G protein-mediated cAMP responses (37). Likewise, carvedilol has been reported to display β-arrestin-biased properties in specific experimental systems, although the magnitude and physiological relevance of this signaling bias remain context dependent (81). Complementary structural studies further demonstrate that subtle modifications of ligand structure can stabilize distinct receptor conformations and selectively alter transducer coupling, providing a molecular basis for pathway-selective β2-AR modulation (37, 82). Although no β2-AR-selective biased agonists have yet entered dedicated clinical trials for myocardial infarction, early first-in-human studies of biased adrenergic ligands in other disease settings support the feasibility of this strategy (83). Future development of pathway-selective or immune cell-targeted β2-AR modulation remains an experimental direction, and its therapeutic value in myocardial injury requires direct validation.

### 5.3 Prospects in gene and cell therapy

Building upon the mechanistic insights discussed throughout this review, emerging gene- and cell-based therapies aim not simply to enhance β2-AR activity but to restore, recalibrate, or selectively reprogram immune-cell β2-AR signaling. These approaches represent a shift from conventional pharmacological receptor activation toward precision immunomodulation based on specific cellular and molecular defects. One promising strategy involves direct restoration of immune-cell β2-AR expression. Experimental studies using lentiviral-mediated gene transfer demonstrated that reconstitution of β2-AR-deficient bone marrow cells before transplantation restored leukocyte mobilization to the injured myocardium, reduced splenic VCAM-1 expression, and significantly improved survival after myocardial infarction (6). These findings provide strong causal evidence that restoration of β2-AR can rescue the phenotype produced by experimental receptor deficiency. However, this rescue experiment should be distinguished from therapeutic efficacy in receptor-intact disease models, and whether β2-AR gene delivery can provide therapeutic benefit after myocardial injury remains to be established. Rather than replacing the receptor itself, an alternative strategy is to selectively manipulate downstream signaling pathways. Annexin A1-associated pro-resolving pathways have been implicated in macrophage efferocytosis, inflammation resolution, and cardiac repair after myocardial injury (60–62). Likewise, increasing attention has focused on biased modulation of β-arrestin signaling. Pharmacological agents capable of preferentially engaging selected β-arrestin-associated pathways while minimizing maladaptive signaling illustrate the feasibility of pathway-selective GPCR modulation and provide an important conceptual framework for future β2-AR-directed therapeutics (84, 85).

Advances in immune-cell engineering further extend these concepts. Ex vivo genetic modification of macrophages or other immune-cell populations to enhance β2-AR expression or optimize downstream signaling may improve leukocyte trafficking, inflammatory resolution, and reparative function following reinfusion. Compared with systemic pharmacological interventions, engineered immune-cell therapies offer the potential for greater cellular specificity while reducing unintended effects on non-immune tissues. At present, however, β2-AR-directed engineering of immune cells remains a proposed strategy rather than an established therapeutic approach in myocardial injury.

Despite these encouraging developments, successful clinical translation will require consideration of patient-specific biological contexts. Metabolic disorders such as diabetes mellitus, obesity, and chronic heart failure profoundly alter β-adrenergic receptor responsiveness and downstream signaling competence (25, 27, 86). Chronic metabolic stress can promote GRK2-associated receptor desensitization (25, 86), expand CCR2+ inflammatory macrophage populations and monocyte recruitment in diabetic cardiac injury (87), and compromise macrophage efferocytosis under diabetic conditions (88), all of which may substantially modify therapeutic responsiveness to β2-AR-targeted interventions. Consequently, future β2-AR-directed therapies are unlikely to follow a “one-size-fits-all” paradigm. Instead, integrating metabolic status, inflammatory phenotype, receptor signaling competence, and disease stage into patient stratification may ultimately support more individualized investigation of immune-cell β2-AR signaling and maximize therapeutic efficacy after myocardial injury.

### 5.4 Personalized therapy and biomarkers

The highly dynamic and cell-specific nature of immune-cell β2-AR signaling suggests that uniform adrenergic modulation is unlikely to achieve optimal therapeutic benefit in all patients. Instead, future β2-AR-targeted therapies may require biomarker-guided patient stratification capable of characterizing receptor signaling status and immune activation, although whether such biomarkers can predict responsiveness to specific adrenergic interventions remains unknown. Among the most promising candidate biomarkers are components of the β2-AR signaling cascade itself. Increased expression of GRK2 in circulating immune cells has consistently been associated with impaired β-adrenergic responsiveness, reduced left ventricular ejection fraction, and adverse outcomes in patients with heart failure (89, 90). These associations support the prognostic relevance of immune-cell GRK2 but do not establish its ability to predict treatment response. Likewise, alterations in the relative expression of β-arrestin 1 and β-arrestin 2 may reflect changes in receptor signaling bias and downstream pathway selection, although clinically validated thresholds have not yet been established (91). Functional assessment of cAMP responsiveness may further complement these molecular markers by providing a direct readout of receptor signaling competence.

Advances in detection technologies are expanding opportunities for dynamic monitoring of immune-cell β2-AR signaling. GRK2 protein levels and activity can be quantified in circulating lymphocytes (89, 90). More recently, liquid biopsy approaches based on circulating extracellular vesicles and exosomes have emerged as attractive minimally invasive platforms for evaluating β2-AR-related signaling signatures and neuroimmune communication (92–94).In parallel, positron emission tomography (PET) imaging may provide complementary information regarding cardiac sympathetic activity and receptor distribution, offering the possibility of integrating systemic immune biomarkers with tissue-level functional imaging.

Despite these advances, several barriers remain before immune-cell β2-AR biomarkers can be incorporated into routine clinical practice. Current assays are relatively labor-intensive, lack standardized analytical protocols, and have not yet demonstrated clear incremental value beyond established biomarkers such as natriuretic peptides or cardiac troponins. Large prospective studies are therefore needed to establish clinically meaningful thresholds, validate prognostic performance, and determine separately whether any of these markers can predict response to specific adrenergic therapies, while also evaluating cost-effectiveness within existing risk-stratification frameworks. Beyond biomarker development, patient-specific biological characteristics are also likely to influence therapeutic responsiveness. Sex hormones modulate β-adrenergic signaling efficiency, receptor desensitization, and sympathetic activation patterns, whereas aging is associated with reduced receptor density, impaired G protein coupling, enhanced GRK-mediated desensitization, and immunosenescence (95–99). Together with metabolic comorbidities such as diabetes and obesity, these factors reshape immune-cell β2-AR signaling and may contribute to substantial inter-individual variability in treatment response.

## 6 Future directions

Current evidence supports immune-cell β2-AR as an important component of neuroimmune regulation during myocardial injury, but several fundamental questions remain unresolved. Apparently divergent findings across experimental studies should not necessarily be interpreted as evidence for a single, established temporal sequence of β2-AR function, particularly because some β2-AR-associated effects, such as suppression of inflammatory cytokine production and facilitation of leukocyte recruitment, are not mutually exclusive and may occur in parallel (11, 14). Instead, differences in the immune-cell populations targeted, genetic strategies employed, injury models, adrenergic stimulation, and local inflammatory environments may all contribute to distinct biological outcomes (6, 7, 60). In particular, the relative contributions of circulating/recruited immune cells and pre-existing cardiac-resident immune populations remain incompletely defined (57, 59). Resolving these sources of heterogeneity will be essential for determining under which conditions and in which cellular compartments β2-AR signaling is protective, maladaptive, or functionally neutral. A proposed temporal framework for investigating immune-cell β2-AR signaling across the overlapping phases of myocardial injury and repair is presented in **Figure 4**.

**Figure 4:**
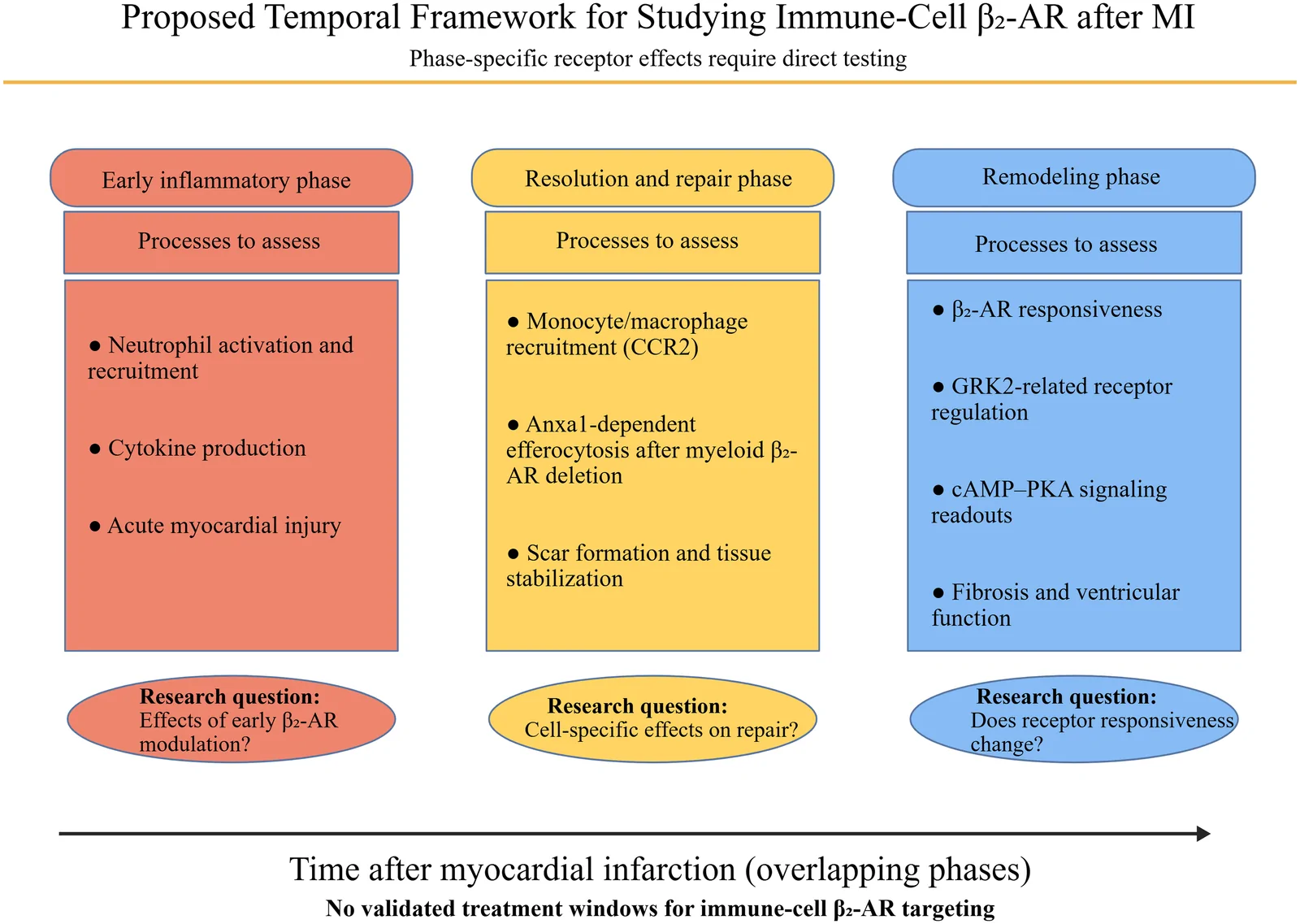
Proposed temporal framework for studying immune-cell β2-AR after myocardial infarction. Overlapping inflammatory, repair, and remodeling phases organize candidate assessments of leukocyte recruitment, cytokine production, efferocytosis, scar formation, receptor responsiveness, and ventricular function. This framework guides experimental investigation; phase-specific receptor effects and therapeutic windows for immune-cell β2-AR targeting remain to be established.

A major limitation of the current literature is that most mechanistic studies rely on global hematopoietic, leukocyte-specific, or broader lineage-restricted models, which do not fully resolve the contributions of individual immune-cell subsets. Future studies should therefore employ lineage-resolved and inducible genetic approaches capable of distinguishing neutrophils, circulating monocytes, recruited macrophages, cardiac-resident macrophages, dendritic cells, and lymphocyte populations. Fate mapping and temporally controlled receptor manipulation may be particularly valuable for separating effects related to cellular origin from those associated with activation state or disease stage.

Another important challenge is the distinction between receptor expression and receptor function. β2-AR abundance alone may not adequately reflect signaling competence because receptor phosphorylation, G-protein coupling, GRK-mediated desensitization, β-arrestin engagement, intracellular compartmentalization, and signaling bias can substantially modify downstream responses (25, 43, 47). Longitudinal functional assessment of these signaling properties, rather than reliance on single time-point measurements of receptor expression, will be necessary to determine how immune-cell β2-AR signaling changes during myocardial injury and repair.

Emerging single-cell multi-omics, spatial transcriptomics, lineage tracing, and high-dimensional immune profiling provide opportunities to address these questions while preserving cellular, spatial, and temporal information (56–59). Integrating these approaches with functional measurements of cAMP responsiveness, GRK activity, β-arrestin engagement, and downstream signaling may enable more precise characterization of β2-AR states within individual immune-cell populations. Such studies should also account for clinically relevant variables, including aging, sex, diabetes, obesity, chronic sympathetic activation, and heart failure, because most existing experimental studies have been conducted in young and otherwise healthy animals.

Finally, translation of immune-cell β2-AR biology into therapy will require a clearer distinction between established cardiovascular effects of adrenergic drugs and immune mechanisms that remain experimentally inferred (75, 77, 78, 100). Future clinical studies should determine whether immune-cell β2-AR status provides information beyond conventional cardiovascular biomarkers and whether it can identify patients who are more likely to benefit from specific adrenergic interventions (89, 90). Until such evidence is available, cell-specific, pathway-selective, and biomarker-guided modulation of β2-AR signaling should be regarded as promising directions for investigation rather than established therapeutic strategies.

## 7 Conclusion

Immune-cell β2-AR provides an important interface between sympathetic signaling and immune responses during myocardial injury. Available evidence indicates that its biological effects are highly dependent on immune-cell identity, anatomical compartment, signaling state, and disease context. Rather than supporting a uniform anti-inflammatory or pro-reparative role, current findings highlight substantial heterogeneity across immune populations and experimental models. Greater resolution of cell-specific signaling mechanisms, particularly the distinction between circulating/recruited and cardiac-resident immune cells, will be essential for defining the therapeutic potential of this pathway. Advances in lineage-resolved models, spatial and single-cell technologies, and functional assessment of receptor signaling may ultimately provide the evidence required to translate immune-cell β2-AR biology into more precise approaches to cardiovascular immunomodulation.

## Glossary

Akt: Protein kinase B
Anxa1: Annexin A1
Arg-1: Arginase-1
cAMP: Cyclic adenosine monophosphate
CCL2: C-C motif chemokine ligand 2
CCR2: C-C chemokine receptor type 2
CREB: cAMP response element-binding protein
CTLA-4: Cytotoxic T-lymphocyte-associated protein 4
EGFR: Epidermal growth factor receptor
ERK1/2: Extracellular signal-regulated kinase 1/2
Foxp3: Forkhead box P3
FPR2/ALX: Formyl peptide receptor 2/lipoxin A4 receptor
GPCR: G protein-coupled receptor
GRK2: G protein-coupled receptor kinase 2
HF: Heart failure
HSPC: Hematopoietic stem and progenitor cell
I/R: Ischemia/reperfusion
IFN-γ: Interferon-γ
IL: Interleukin
iNOS: Inducible nitric oxide synthase
IκB: Inhibitor of κB
MAPK: Mitogen-activated protein kinase
MCP-1: Monocyte chemoattractant protein-1
MI: Myocardial infarction
NETs: Neutrophil extracellular traps
NF-κB: Nuclear factor kappa B
NK: Natural killer
PET: Positron emission tomography
PI3K: Phosphoinositide 3-kinase
PKA: Protein kinase A
ROS: Reactive oxygen species
STEMI: ST-segment elevation myocardial infarction
TCR: T-cell receptor
TGF-β: Transforming growth factor-β
Th1: T helper 1
Th17: T helper 17
Th2: T helper 2
TNF-α: Tumor necrosis factor-α
Treg: Regulatory T cell
VCAM-1: Vascular cell adhesion molecule-1
β1-AR: β1-adrenergic receptor
β2-AR: β2-adrenergic receptor
βARKct: β-adrenergic receptor kinase C-terminal peptide.
